# Transcriptome sequencing and analysis during seed growth and development in *Euryale ferox* Salisb

**DOI:** 10.1186/s12864-018-4707-9

**Published:** 2018-05-09

**Authors:** Xian Liu, Zhen He, Yulai Yin, Xu Xu, Weiwen Wu, Liangjun Li

**Affiliations:** 1 School of Horticulture and Plant Protection, Yangzhou University, 48 Wenhui East Road, Yangzhou, Jiangsu Province 225009 People’s Republic of China; 2 Suzhou Vegetable Research Institute, 188 Xitang Road, Suzhou, Jiangsu Province 215008 People’s Republic of China

**Keywords:** *Euryale ferox* Salisb., Seed development, Transcriptome, Phenylpropanoid biosynthesis, Phenylalanine ammonia-lyase, Cytochrome P450

## Abstract

**Background:**

*Euryale ferox* Salisb., an annual aquatic plant, is the only species in the genus *Euryale* in the Nymphaeaceae. Seeds of *E. ferox* are a nutritious food and also used in traditional Chinese medicine (Qian Shi in Mandarin). The molecular events that occurred during seed development in *E. ferox* have not yet been characterized. In this study, we performed transcriptomic analysis of four developmental stages (T1, T2, T3, and T4) in *E. ferox* seeds with three biological replicates per developmental stage to understand the physiological and biochemical processes during *E. ferox* seeds development.

**Results:**

313,844,425 clean reads were assembled into 160,107 transcripts and 85,006 unigenes with N50 lengths of 2052 bp and 1399 bp, respectively. The unigenes were annotated using five public databases (NR, COG, Swiss-Prot, KEGG, and GO). In the KEGG database, all of the unigenes were assigned to 127 pathways, of which phenylpropanoid biosynthesis was associated with the synthesis of secondary metabolites during *E. ferox* seed growth and development. Phenylalanine ammonia-lyase (PAL) as the first key enzyme catalyzed the conversion of phenylalanine to trans-cinnamic acid, then was related to the synthesis of flavonoids, lignins and alkaloid. The expression of *PAL1* reached its peak at T3 stage, followed by a slight decrease at T4 stage. Cytochrome P450 (P450), encoded by *CYP84A1* (which also called ferulate-5-hydroxylase (F5H) in Arabidopsis), was mainly involved in the biosynthesis of lignins.

**Conclusions:**

Our study provides a transcriptomic analysis to better understand the morphological changes and the accumulation of medicinal components during *E. ferox* seed development. The increasing expression of PAL and P450 encoded genes in phenylpropanoid biosynthesis may promote the maturation of *E. ferox* seed including size, color, hardness and accumulation of medicinal components.

**Electronic supplementary material:**

The online version of this article (10.1186/s12864-018-4707-9) contains supplementary material, which is available to authorized users.

## Background

**Fig. 1:**
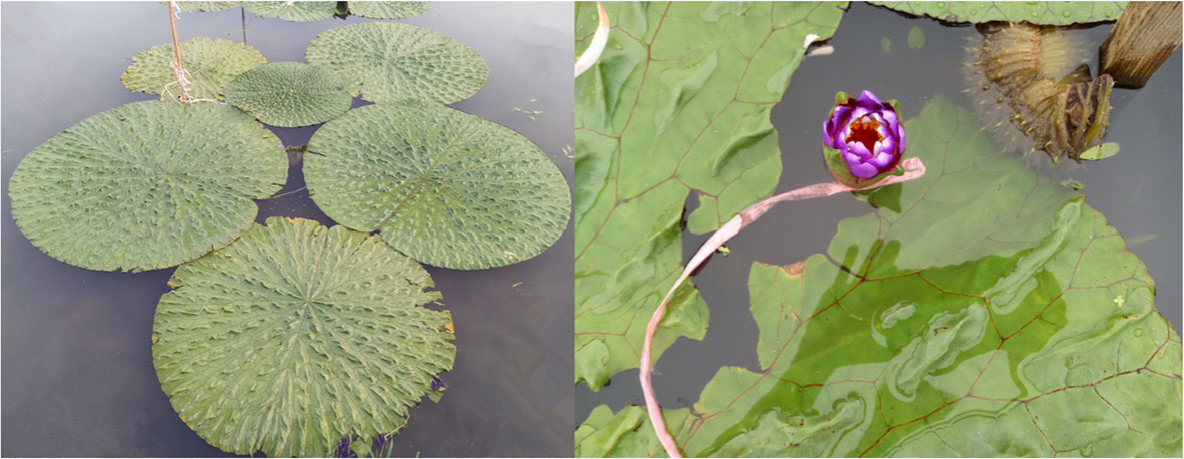
The leaves and flower of *Euryale ferox* Salisb. in shallow body of water

*Euryale ferox* Salisb., an annual aquatic herbaceous plant, is the only species in the genus *Euryale* in the botanical family Nymphaeaceae [1, 2]. *E. ferox* is known commonly as fox nut or gorgon nut [3], and widely distributed in tropical and subtropical regions of east and southeast Asia [4]. *E. ferox* is usually grown in lakes, ponds, reservoirs, and other shallow bodies of water [4, 5]. Unlike most other aquatic crops, the floating leaves of *E. ferox* are comparatively large, reaching up to 1.5 m in diameter [5] (Fig. 1). The seeds of *E. ferox* are rich in starch, proteins, vitamins, minerals and many other nutritional ingredients [6, 7]. The “Compendium of Materia Medica”, a book of Chinese traditional medicine written during the Ming dynasty (1368–1644), describes the many medicinal uses of *E. ferox*, which included its use as a kidney tonic, for nourishing the spleen, as a treatment for diarrhea, as well as for eliminating dampness [8]. Seeds of *E. ferox* are also a significant component of contemporary Chinese herbal medicine and are used to treat a variety of diseases, such as kidney failure, chronic diarrhea, excessive leucorrhea, and hypofunction of the spleen [9, 10]. At present, studies of *E. ferox* are focused on seed dormancy and germination traits [4], genetic diversity [11], and the antioxidant activity in the seeds [3, 10, 12, 13]. However, there are few reports describing the stages of seed development in *E. ferox* in the literature. It is particularly important for seeds to explore the morphological changes and the accumulation of medicinal components.

Phenylpropanoid biosynthesis involved in numerous important biological processes, such as detoxification of xenobiotics and synthesis of secondary metabolites [14, 15], plays an important role in development of many plant. Phenylalanine ammonia-lyase (PAL) [14] and cytochrome P450 (P450) [16] both encoded by a multi-gene family with different functions [17–22] are key enzymes in the proceed of phenylpropanoid biosynthesis. The expression of *PALs* possesses tissue-specific in *Arabidopsis thaliana* [23] and *Salvia miltiorrhiza* [14], among which the highest expression of *PAL1* and *PAL2* in root and mature flower of *Arabidopsis thaliana* [23], *PAL1*, *PAL2* and *PAL3* with highest expression in root, stem and leaf of *Salvia miltiorrhiza*, respectively [14]. The expression of *FaPAL6* increased during the strawberry fruit riping along with the anthocyanin accumulation affecting fruit color [24]. For *P450* genes (*cytochrome P450*, *CYP*), the expression of *CaCYP94B1* was up-regulated, whereas the expressions of *CaCYP72A15*, *CaCYP701A3* and *CaCYP71A25* were down-regulated in the perisperm at three developmental stages of *Coffea Arabica* [25]. In addition, *TaCYP78A3* was specifically expressed in reproductive organs and influenced the size of wheat seed [26]. *E. ferox* seed, as an important medicinal component, was closely related to many secondary metabolites, such as flavonoids, lignins and alkaloid [14]. Phenylpropanoid biosynthesis involved in the synthesis of secondary metabolites was selected to analyse the relationship with *E. ferox* seed development, and *PAL* and *P450* are the vital differential genes in this pathway that we found.

In this study, we performed a transcriptomic analysis of *E. ferox* seed at four developmental stages (10, 20, 30, and 40 days after anthesis) on the basis of morphological characteristics [27, 28]. Quantitative real-time PCR on the basis of six genes associated with nutritional and functional substances of *E. ferox* seed validated the transcriptome data. Two unigenes –PAL and P450 encoded genes associated with phenylpropanoid biosynthesis during *E. ferox* seed development were analysed to investigating the relationship with morphological changes and the accumulation of medicinal components.

## Methods

### Plant material and sample collection

**Fig. 2:**
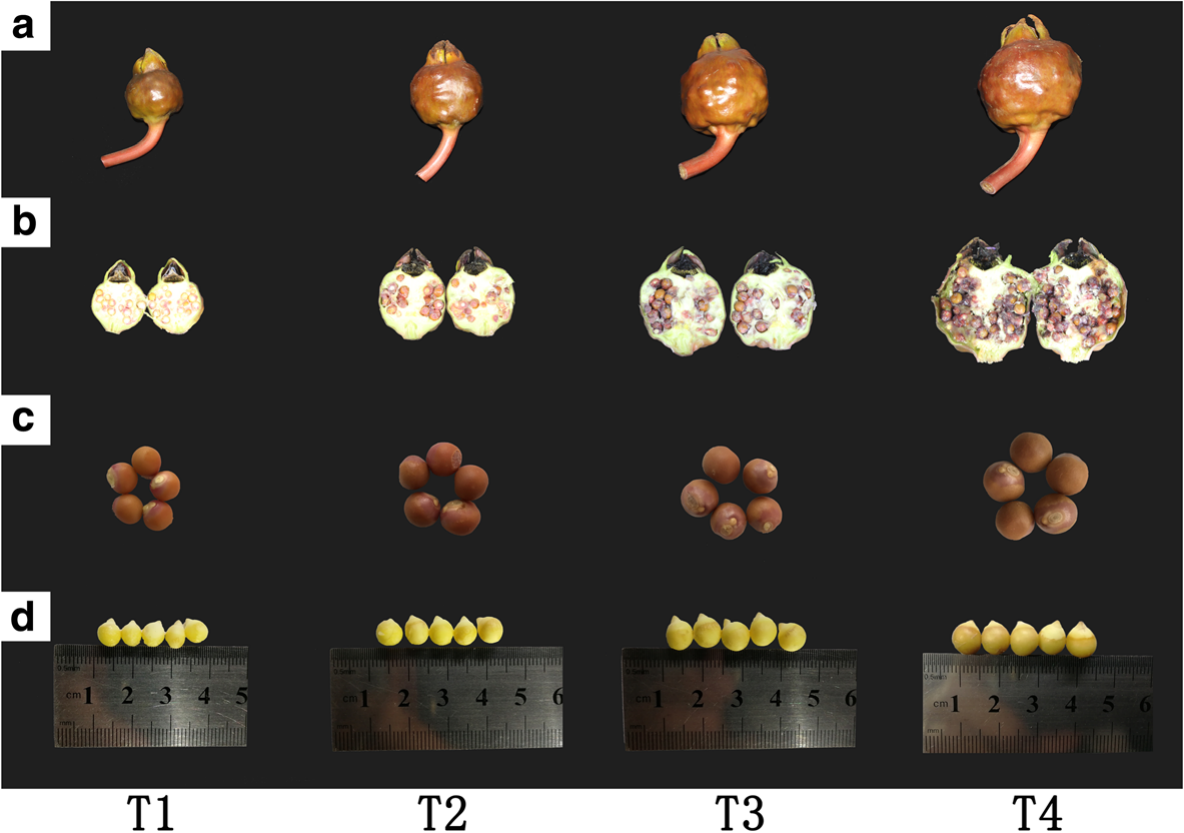
The fruit, the longitudinal section of fruit, seed and kernel of *Euryale ferox* Salisb. during four development stages (T1, T2, T3 and T4). **a** Fruit; (**b**) The longitudinal section of fruit; (**c**) Seed; (**d**) Kernel

*Euryale ferox* plants were collected from Suzhou of Jiangsu Province, China. Specifically, seeds of *E. ferox* begin to swell at 10 days after anthesis, and grow rapidly from 20 to 30 days after anthesis, while reach physiological maturity at 40 days after anthesis (Fig. 2). So the seed samples used for cDNA library construction were collected on 10, 20, 30, and 40 days after anthesis (stages T1, T2, T3, and T4, respectively), with three biological replicates per developmental stage. All samples were frozen in liquid nitrogen immediately upon collection and stored at − 80 °C.

### RNA preparation and cDNA library construction

Total RNA was extracted from the 12 samples separately. RNA contamination and degradation was detected by electrophoresis on 1% agarose gels. RNA purity was measured with a NanoPhotometer® spectrophotometer (IMPLEN, CA, USA), and RNA concentration was determined with a Qubit®2.0 Flurometer and the Qubit® RNA Assay Kit (Life Technologies, CA, USA). RNA integrity was evaluated using the Nano 6000 Assay Kit on the Agilent Bioanalyzer 2100 system (Agilent Technologies, CA, USA).

To construct the cDNA libraries, we used 3 μg total RNA per sample as the input material. The NEBNext® Ultra™ RNA Library Prep Kit for Illumina® (NEB, USA) was used to generate a series of corresponding libraries, and index codes were applied to every sample. Briefly, mRNA was purified from total RNA using oligo dT magnetic beads. RNA fragmentation was carried out at high temperature in the presence of divalent cations in NEBNext First Strand Synthesis Reaction Buffer (5X). First-strand cDNA was synthesized using random hexamer primers and M-MLV Reverse Transcriptase (RNase H^−^). Second strand was synthesized using DNA Polymerase I and RNase H. The exonuclease/polymerase activities were used to convert the remaining single-stranded overhangs to blunt ends. After adenylation of the 3′ ends of the DNA fragments, the NEBNext Adaptor containing a hairpin loop structure was added. cDNA fragments between 150 and 200 bp were purified using the AMPure XP system (Beckman Coulter, Beverly, USA), and the cDNA library was non-strand specific. Afterwards, the 3 μl USER Enzyme (NEB, USA) was made use of picking size, adaptor-ligated cDNA at 37 °C for 15 min followed by 5 min at 95 °C before PCR. PCR was performed using universal PCR primers, Index (X) Primer and Phusion High-Fidelity DNA polymerase. The Agilent Bioanalyzer 2100 system was used to purify the PCR products and to evaluate the quality of the cDNA libraries. Paired-end DNA sequencing data for the 12 libraries was generated on an Illumina Hiseq 2000 instrument.

### De novo cDNA assembly and functional annotation

To obtain clean reads, sequencing adaptors and low-quality reads were removed from each library. For increasing the amount of date to reduce the unigene redundancy, and ensuring the low expression genes assembled more completely, the remaining clean reads were adopted the method of the merged assembly of Trinity software [29]. Specifically, the clean reads in each library were first assembled into contigs, then the contigs were clustered to form unigenes. The clean reads were mapped back to the homologous contigs based on the paired-end reads, and the different contigs from their transcripts and the distance were also distinguished by the methods of the De Bruijn and sequencing information.

The unigene sequences were annotated using the following public databases: NCBI non-redundant protein sequences (NR) [30], Clusters of Orthologous Groups (COG) [31], Swiss-Prot [32], Kyoto Encyclopedia of Genes and Genomes (KEGG) [33], and Gene Ontology (GO) [34]. The unigene sequences were aligned using BLASTX with an E-value of < 10^− 5^. If results from the different databases were mutually contradictory, a priority order of NR, Swiss-Prot, KEGG, and COG was followed to decide the annotation. Nevertheless, if a unigene did not align to any of the databases, ESTScan software was chosen to infer the direction of transcription [35].

### Differential gene expression analysis

Each of the 12 *E. ferox* seed cDNA libraries was aligned separately to the transcriptome assemblies using Bowtie [36]. The counting of alignments was estimated using the RSEM package [37]. The RPKM method was then used to calculate the numbers of differentially expressed genes (DEGs) from pairwise comparisons of the four seed developmental stages [38]. From the biological replicates in this study, DESeq [39] implemented in R package was used to analyze the differential expression between two groups. *P* values were adjusted using the Benjamini-Hochberg approach to control the false discovery rate (FDR). Genes with an adjusted *P*-value < 0.05 found by DESeq were usually identified as DEGs. However, genes with FDR < 0.01 and FC (Fold Change) ≥2 served as standards in the screening, and FC represents the specific value between two samples. In addition, the phylogenetic relationships of P450 and beta-glucosidase genes were assessed using the neighbor-joining (NJ) method in MEGA version 7.0 [40].

### Quantitative real-time PCR analysis

Total RNA was extracted in triplicate from the four developmental stages of *E. ferox* seeds (total of 12 samples) using Trizol® Reagent (Invitrogen, USA), and then reversed transcribed into cDNA using a PrimeScript® RT reagent Kit (Takara, Dalian, China). Six differentially expressed genes associated with nutritional and functional substances in *E. ferox* seed were selected to validate the transcriptome data using quantitative real-time PCR. The gene-specific primers for the six unigene sequences were designed with Primer Premier 5 software [41], and the *β*-*Actin* gene was used as an internal gene expression control: the gene was amplified with forward primer 5′-GACTCTGGTGATGGTGT-3′ and reverse primer 5′-CACTTCATGATGGAGTTGT-3′. The amplifications were performed in 20 μl reactions consisting of 10 μl 2× SYBR® *Premix EX* Taqll (Tli RNaseH Plus) (TaKaRa, Dalian, China), 2.0 μl of a mixture of the forward and reverse primers, 2.0 μl cDNA template, and 6.0 μl sterile dH_2_O. The amplifications were performed on a CFX-96 real-time PCR system (Bio-Rad) using the following quantitative real-time PCR program: 95 °C for 3 min, followed by 39 cycles of 95 °C for 30 s and Tm for 30 s. Each amplification reaction was performed in triplicate.

## Results

### Collection of *Euryale ferox* seed

Transcriptome sequencing was used to analyze the complex physiological and biochemical processes that occurred during four developmental stages of *Euryale ferox* seeds. The first stage was designated as 10 days after anthesis with obvious small size fruit and seeds. The other three stages were 20, 30, and 40 days after anthesis. The fruit and seeds gradually became larger along with the color deepening and mechanical hardening of seeds (Fig. 2). Because three biological replicates were carried out for each developmental stage, a total of 12 samples were used in the analysis of seed development. Specific information of 12 samples is shown in Table 1.

**Table 1:** Summary of sample information and transcriptome sequencing output statistics for the *E. ferox* seed RNA-seq libraries

Sample	Date of Collection	Total Clean Reads	Total Clean Nucleotides (nt)	GC Percent (%)	N Percent (%)	Q30 Percent (%)
1–1	20, August, 2016	22,421,428	6,651,313,472	49.27	0	91.98
1–2	20, August, 2016	28,358,306	8,449,902,688	49.06	0	91.69
1–3	20, August, 2016	21,297,839	6,334,599,486	49.00	0	91.71
2–1	30, August, 2016	24,816,321	7,393,759,062	48.80	0	91.18
2–2	30, August, 2016	25,714,239	7,656,559,222	49.68	0	92.62
2–3	30, August, 2016	23,400,093	6,976,232,182	49.94	0	91.10
3–1	09,September, 2016	22,236,747	6,619,274,594	50.96	0	91.87
3–2	09,September, 2016	24,450,792	7,274,216,700	50.79	0	92.11
3–3	09,September, 2016	27,901,420	8,307,711,474	49.20	0	92.09
4–1	19,September, 2016	28,587,601	8,504,756,010	50.28	0	92.56
4–2	19,September, 2016	29,452,392	8,769,841,382	50.83	0	92.16
4–3	19,September, 2016	35,207,247	10,466,146,780	50.73	0	92.42

### RNA-seq and de novo transcriptome assembly

In order to obtain comprehensive and effective transcriptomic information for *E. ferox* seed growth and development, 12 cDNA libraries were constructed and generated paired-end sequence reads using the Illumina Hiseq 2000 platform. Detailed information about the transcriptome sequencing is given in Table 1. A total of 313,844,425 clean reads originated from the 12 *E. ferox* cDNA libraries were obtained following removal of adaptors and low-quality reads from the libraries (the transcriptome data is being submitted to SRA database). The percent G + C, percentage of Ns (unknown bases), and fraction of bases with quality scores of Q30 for the 12 libraries averaged 48.8%, 0%, and 91.1%, respectively. These numbers showed that the data was of high quality. Trinity software was then used to assemble the clean reads into 160,107 transcripts with N50 length of 2052 bp and 85,006 unigenes with N50 length of 1399 bp. The relative lengths of the assembled sequences is one standard by which a transcriptome assembly can be assessed. For the present study, the summary statistics of the assembled transcripts and unigenes are shown in Table 2. Of these, assembled sequences < 200 bp were not taken into account. In the final assemblies there were 60,836 transcripts ranging from 200 to 500 bp in length, which accounted for 38% of the total. There were 32,751 (20.46%) and 66,519 (41.55%) transcripts from 500 to 1000 bp and > 1000 bp in length, respectively. Compared with the assembled transcripts, the assembled unigenes in the above three length ranges accounted for 59.91%, 19.16%, and 20.94% of the total (Table 2). In addition, the assembled sequences were also annotated by BLASTX against *Arabidopsis thaliana* TAIR10 (Additional file 3: Table S1) and rice 7.0 proteomes (Additional file 4: Table S2).

**Table 2:** Summary statistics for the assembled transcripts and unigenes

Length range	Transcripts	Transcripts percent (%)	Unigenes	Unigenes percent (%)
200–300	34,006	21.24%	30,871	36.32%
300–500	26,830	16.76%	20,051	23.59%
500–1000	32,751	20.46%	16,287	19.16%
1000–2000	35,975	22.47%	10,154	11.95%
2000+	30,544	19.08%	7642	8.99%
Total number	160,107		85,006	
Total length	193,603,846		66,000,704	
N50 length	2052		1399	
Mean length	1209.22		776.42	

### Annotation and functional classification

**Fig. 3:**
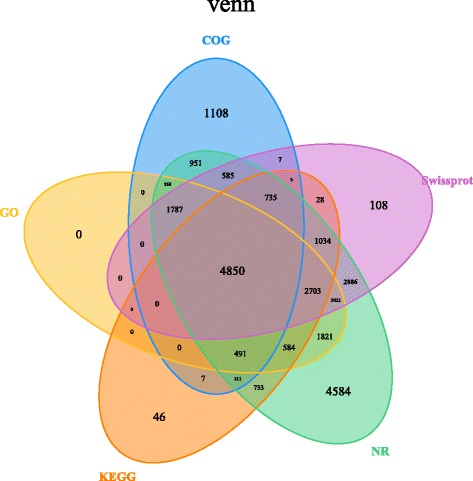
Venn diagram of *Euryale ferox* Salisb. seed unigenes. Functional annotation was done using five public databases; NCBI non-redundant protein sequences (NR), Swiss-Prot, COG, Gene Ontology (GO), and Kyoto Encyclopedia of Genes and Genomes (KEGG). The shared unigenes are indicated in the intersections

The assembled sequences were used as queries in BLAST searches (E ≤ 10^− 5^) against the NR, COG, Swiss-Prot, KEGG, and GO databases, and the results are shown in Fig. 3. The majority of the unigenes (27,546; 32.4%) were annotated from the NR database. In contrast, only 11,304 annotated unigenes were matched to the COG database. As shown in Fig. 3, a total of 4850 unigenes were annotated with all five databases.

**Fig. 4:**
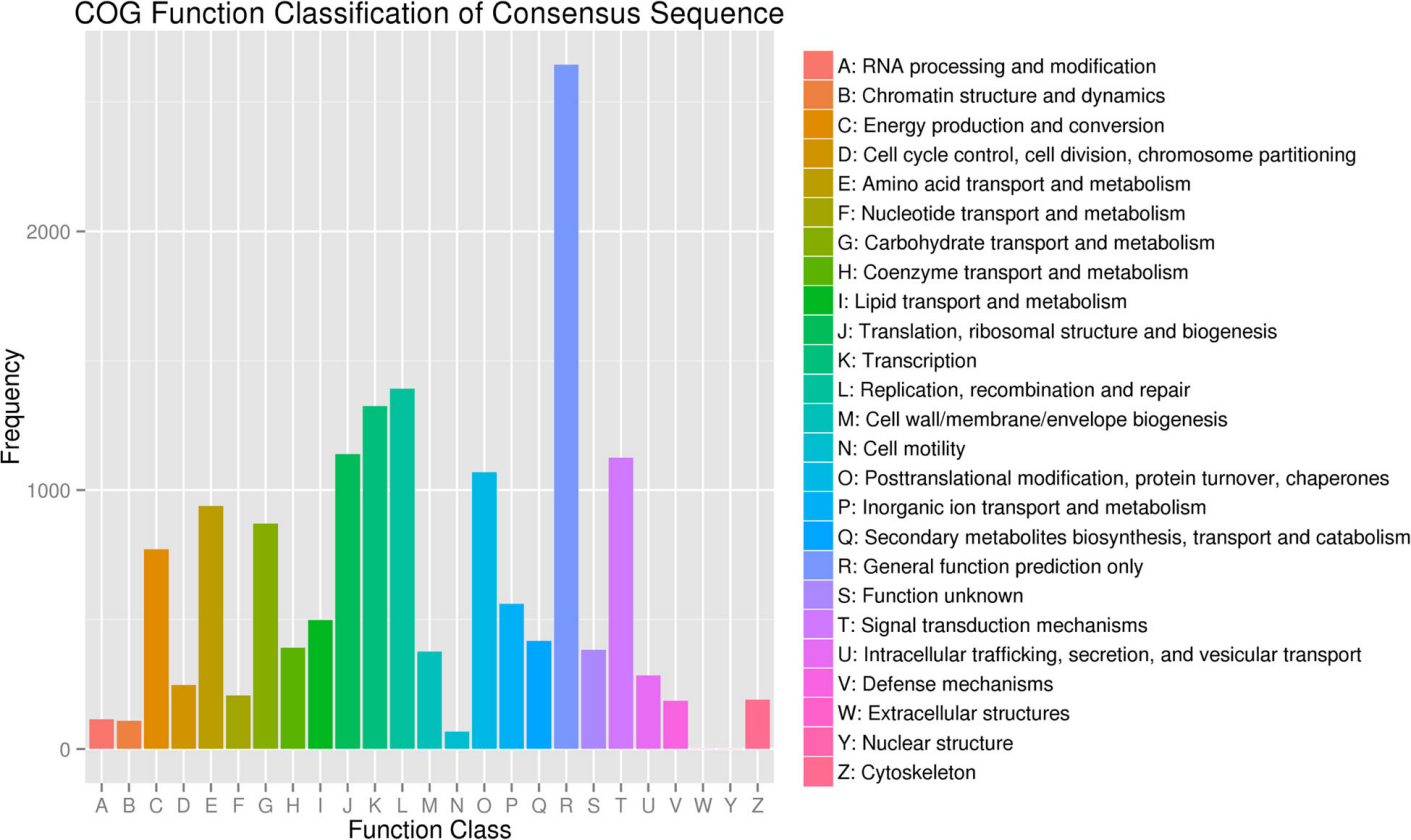
Histogram of clusters of orthologous groups (COG) classification. A total of 11,304 unigenes with lengths more than 300 bp were divided into 25 COG categories

**Fig. 5:**
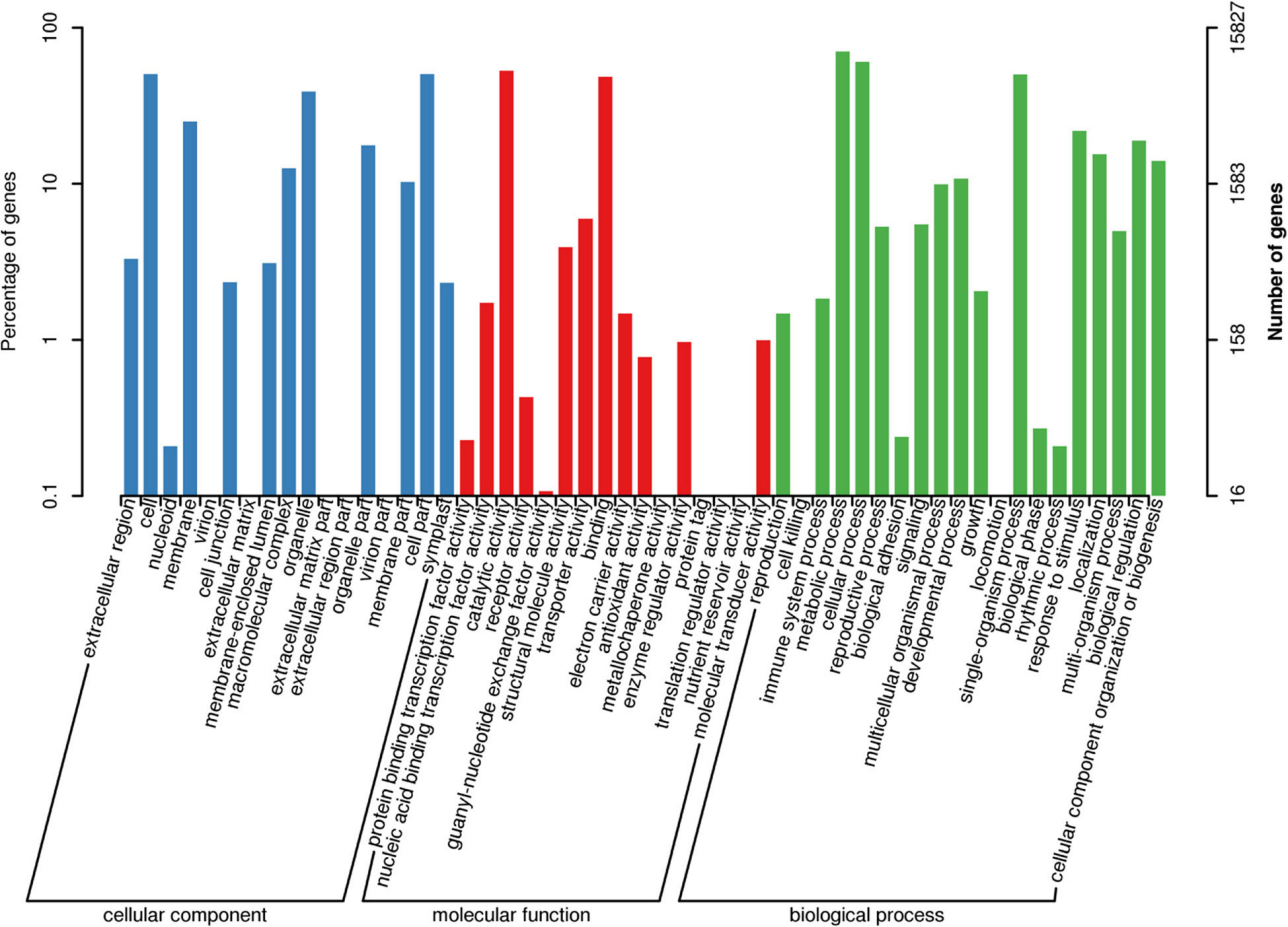
Functional annotation of assembled sequences based on gene ontology (GO) categorization. 15,827 unigenes were grouped into the three main GO domains: “cellular component”, “molecular function”, and “biological process”

To further utilize the transcriptome data, the COG, GO, and KEGG databases were used to identify the molecular processes that occur during the growth and development of *E. ferox* seed. The annotated unigenes were initially classified into 25 COG categories with the largest group being “General function prediction only” (2644; 23.39%) (Fig. 4**)**. Genes in “Carbohydrate transport and metabolism” (870, 7.70%) and “Secondary metabolites biosynthesis, transport and catabolism” (417, 3.69%) are involved in biosynthesis of the nutritional and functional substances which benefit human health. The Gene Ontology (GO), which is an internationally standardized classification system, was used to assign 15,827 unigenes from the four seed developmental stages to the three principal GO domains; “cellular component”, “molecular function”, and “biological process” (Fig. 5). In the “cellular component” category, “cell” and “cell part” were the most abundant terms. For the category of “molecular function”, “catalytic activity” and “binding” were the most prominent. “Metabolic process” and “cellular process” were the most abundant terms in the “biological process” category.

### Differential gene expression and pathway enrichment analysis

Transcriptome sequencing is a powerful and effective approach that is often used to analyze gene expression at the whole genome level without a reference genome sequence, and this method can also be taken to identify a variety of metabolic processes [42–44]. In this study, a total of 4897 DEGs were confirmed among the four developmental stages of *E. ferox* seed. Specific information describing the up- and down-regulated DEGs identified in pairwise comparisons between the four seed developmental stages is shown in Additional file 5: Table S3. The KEGG database is used to systematically map unigenes to specific metabolic pathways and to describe the function of the unigenes. In the present study, all of the unigenes were assigned to 127 pathways which were divided into five groups: cellular processes, environmental information processing, genetic information processing, metabolism, and organismal systems (Additional file 1: Figure S1). Of the five groups, the metabolism group contained the largest number of pathways. The most abundant pathway was “ribosome” which had 539 unigenes, followed by “carbon metabolism” with 515 genes, and “biosynthesis of amino acids” with 470 genes.

### Phenylpropanoid pathway

**Fig. 6:**
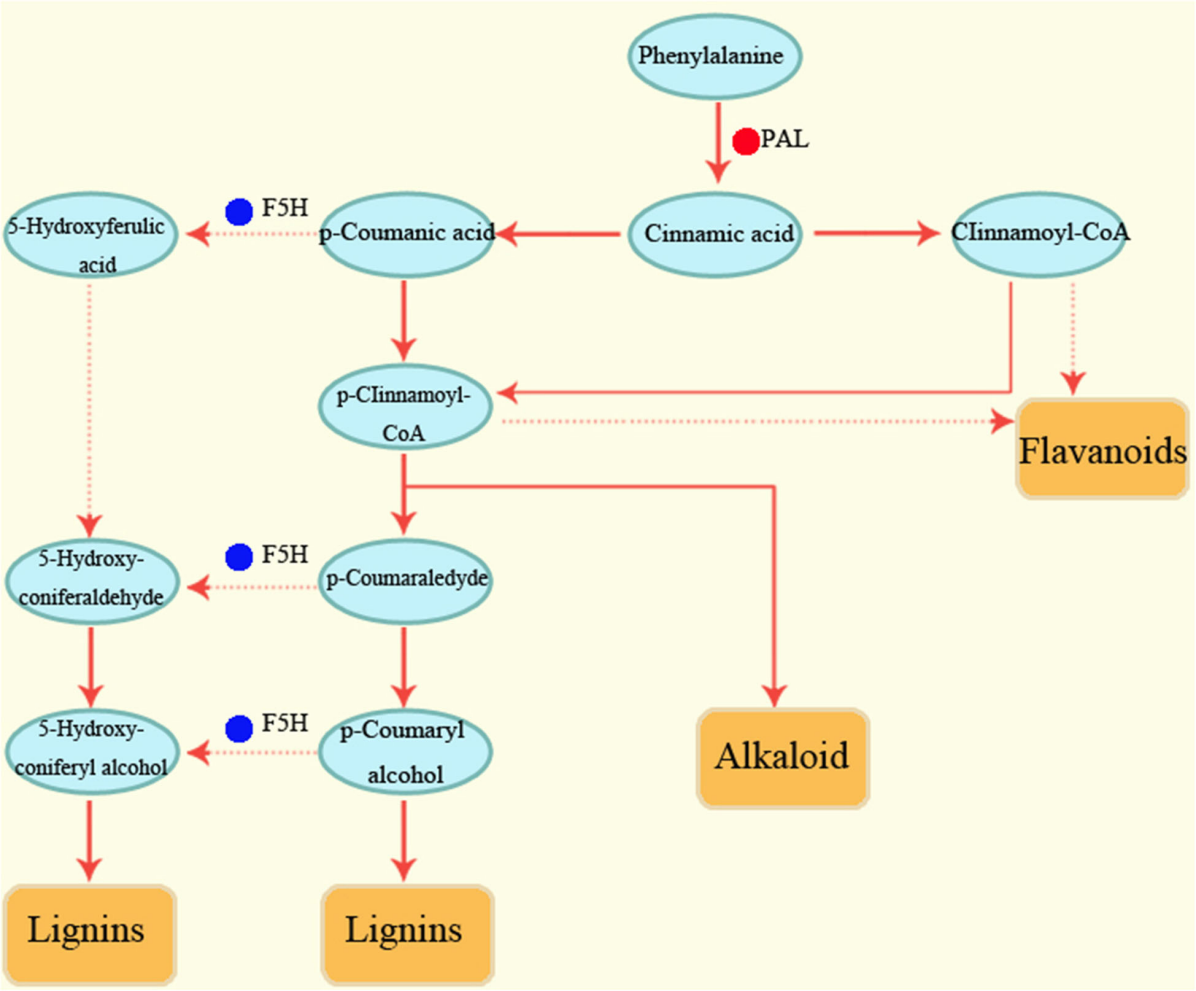
Simplified scheme of phenylpropanoid biosynthesis. Two key enzymes are PAL (phenylalanine ammonia-lyase) and F5H (ferulate-5-hydroxylase) which called the *CYP84A1* gene of cytochrome P450

**Fig. 7:**
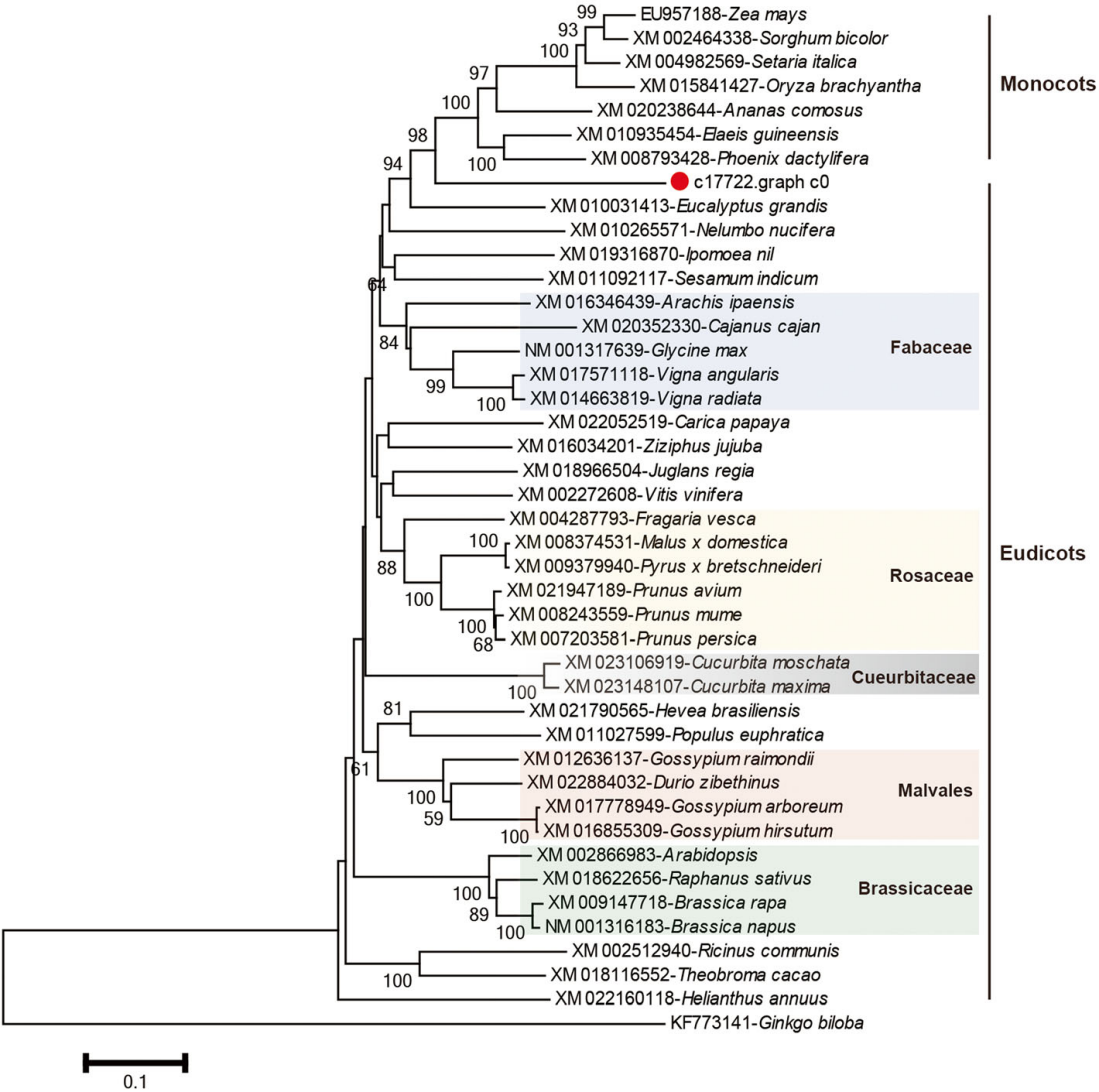
A phylogenetic tree depicting the relationships among the *CYP84A1s* in angiosperm. Forty-two *CYP84A1* genes from angiosperms with *Ginkgo biloba* as a outgroup were used in this study

In the phenylpropanoid pathway of *E. ferox* seed development, PAL is the first key enzyme that catalyzed the conversion of phenylalanine to trans-cinnamic acid, then experienced a series of complex channels to finally synthesizes some important secondary metabolites including flavonoids, lignins and alkaloid (Fig. 6). PAL in *E. ferox* seed was encoded by c55946.graph_c2, which shared highest identities (79%) with *PAL1* gene of Myrtaceae in GenBank. During *E. ferox* seed growth and development, the expression of c55946.graph_c2 reached its peak at T3 stage, followed by a slight decrease at T4 stage. Another key enzyme is P450 encoded by c17722.graph_c0, which shared highest identities (70%) with *CYP84A1* gene of *Ananas comosus* in GenBank*.* And the *CYP84A1* gene was also called ferulate-5-hydroxylase (F5H) in Arabidopsis [45]. In the phenylpropanoid pathway, F5H mainly involved in the biosynthesis of 5-Hydroxy-guaiacyl lignin and syringyl lignin (Fig. 6). Phylogenetic analysis based on P450 gene *CYP84A1* of angiosperms showed that *E. ferox* clustered as the boundary of monocots and eudicots (Fig. 7). The P450 gene sequences of *Zea mays*, *Sorghum bicolor*, *Ananas comosus*, *Phoenix dactylifera*, *Elaeis guineensis*, *Setaria italica*, *Oryza sativa*, *Vitis vinifera*, *Theobroma cacao*, *Glycine max*, *Prunus avium*, *Cucurbita maxima*, *Durio zibethinus*, *Arabidopsis*, *Nelumbo nucifera* etc. in angiosperms together with *Ginkgo biloba* in gymnosperm as outgroup were used in this study. Consistently with P450 gene, the NJ tree based on beta-glucosidase gene showed similar result in Additional file 2: Figure S2.

### Verification of differential gene expression by quantitative real-time PCR

**Fig. 8:**
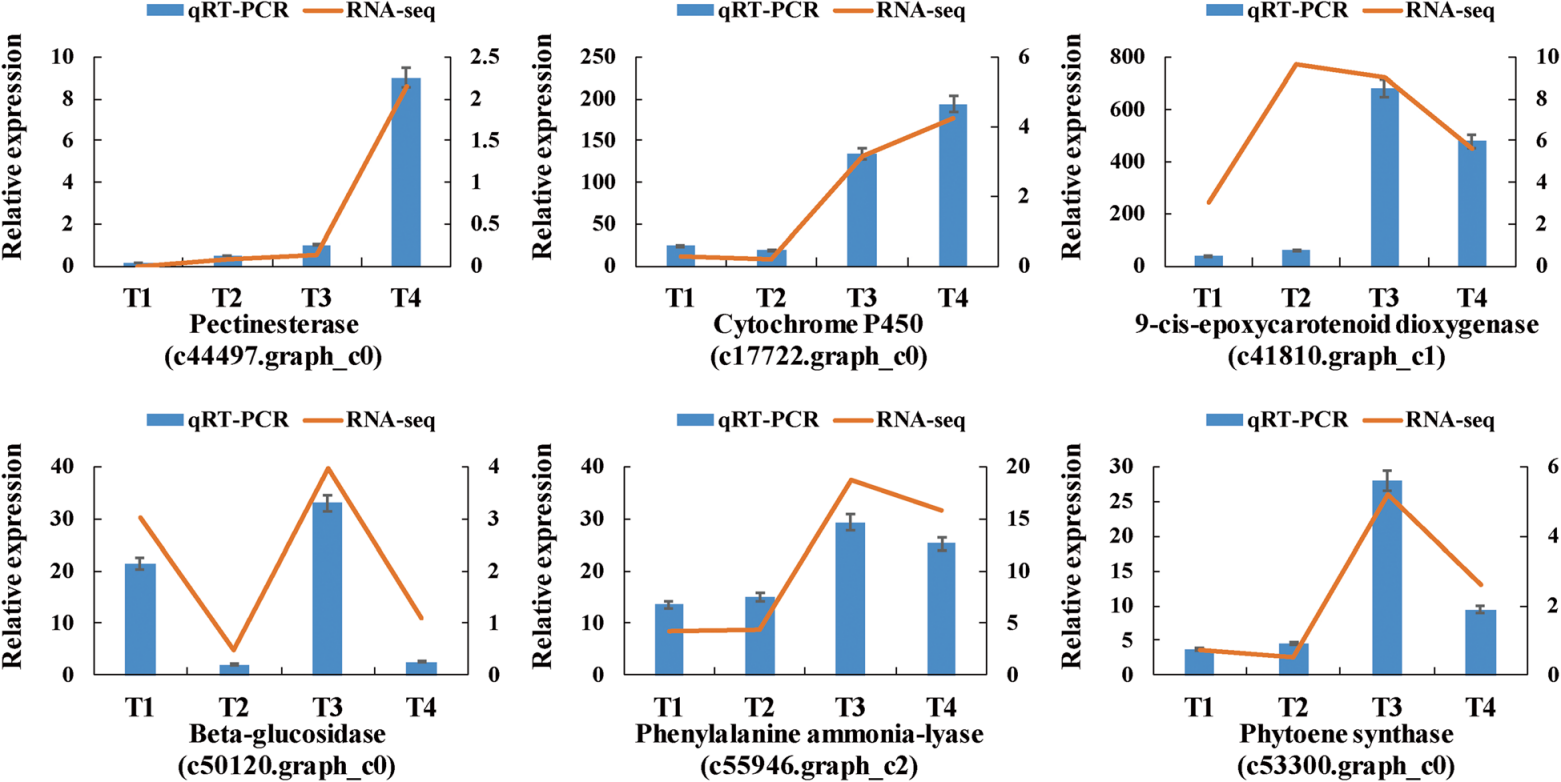
Relative expression levels of six DEGs in four developmental stages of *Euryale ferox* Salisb. seed. We compared expression levels determined by RNA-seq and qRT-PCR analyses for each DEG

To validate the gene expression results derived from the transcriptome data, six DEGs related to nutritional and functional compounds produced during the growth and development of *E. ferox* seed were selected for qRT-PCR. Detailed information about the six DEGs is given in Table 3. These DEGs are mainly involved in “starch and sucrose metabolism” (pectinesterase and beta-glucosidase), “phenylpropanoid biosynthesis” (cytochrome P450 and phenylalanine ammonia-lyase), and also “carotenoid biosynthesis” (9-cis-epoxycarotenoid dioxygenase and phytoene synthase). As shown in Fig. 8, the six DEGs had very similar expression patterns based on the transcriptome data and qRT-PCR results, which indicates that the results of the transcriptomic analysis are reliable (Additional file 6: Table S4).

**Table 3:** Oligonucleotide primers used in gene-specific qRT-PCR assays for six DEGs

Gene identifier	Gene description	Function	Forward primer (5′-3′)	Reverse primer (5′-3′)	Tm (°C)	Product (bp)
c44497.graph_c0	Pectinesterase	Starch and sucrose metabolism	CGCTGAAGTTTTGGGAGATG	CCGACCTCCATTAGAAAACG	54.9	113
c50120.graph_c0	Beta-glucosidase	Starch and sucrose metabolism	GAACCCTCTTGAGCCTACTTGG	TCCATCCACCGCACTGATAACC	60.6	185
c17722.graph_c0	Cytochrome P450	Phenylpropanoid biosynthesis	TCTCGGTGATGAGTCCTCTGCT	GTTCCGCCATTGCCCATTCTAT	60.6	157
c55946.graph_c2	Phenylalanine ammonia-lyase	Phenylpropanoid biosynthesis	CTTCTCTGGCATTCGGTTCG	ACGATAGCGGCACCAAGTCC	60.6	121
c41810.graph_c1	9-cis-epoxycarotenoid dioxygenase	Carotenoid biosynthesis	GTCATCCGAAAGCCTTACCTCC	GTGGAAGCAGAAGCAGTCAGGG	60.6	293
c53300.graph_c0	Phytoene synthase	Carotenoid biosynthesis	CCTGTTGACATCCAGCCATT	TCATCAGACCGACAGTTCCA	55.6	129

## Discussion

In recent years, gene expression in an increasing number of horticultural plants has been studied by transcriptomic analysis including cucumber, tomato, water chestnut, lettuce, and peanut [27, 46–49]. With the rapid development of sequencing technology, transcriptomic analysis has become more efficient and cost-effective. Liu et al. [48] reported that 51,792 unigenes with an average length of 849 bp and an N50 length of 1448 bp were obtained from lettuce seed using Trinity assembly software. Yin et al. [49] assembled 59,236 unigenes with a mean length of 751 bp and an N50 length of 1130 bp from transcriptome sequencing of peanut seed. In the present study, the N50 length from our *E. ferox* seed transcriptome assemblies was greater than in peanut seed but less than in lettuce seed, and all three were generated on the Illumina Hiseq 2000 platform [48, 49]. Undoubtedly, the transcriptome data differs between the three horticultural plant species. In addition, being edible seeds, over 1500 unigenes participate in lipid metabolism in the seven pathways that are related to the accumulation of oil in peanut seed during development [49]. We identified 313 unigenes in the four pathways involved in lipid metabolism in *E. ferox* seed development. This difference must be due to individual crop attributes; in this case, peanut is an oil crop, and the seeds are rich in oil, but *E. ferox* seed is mainly composed of carbohydrates.

PAL is the key enzyme encoded by a multi-gene family in the biosynthesis of many valuable secondary metabolites, including flavonoid, alkaloid and lignin. There are four members in Arabidopsis [18] and tobacco [19], seven members in cucumber [17], and more than twenty copies in tomato [20]. In this study, gene encoding PAL of *E. ferox* seed is grouped into *PAL1*. *PAL1* has been extensively studied in other plants in order to observe tissue-specific expression and understand secondary metabolites [14, 17–19]. The expression of *PAL1* in *E. ferox* seed reached its peak at T3 period, followed by a slight decrease at T4 period. The increasing expression patterns during the maturity of seed influenced the increase of relevant secondary metabolites in phenylpropanoid pathway, which may explain the morphological changes and the accumulation of medicinal substances in *E. ferox* seed. Similar, in the development of the apple (variety ‘Jonathan’) and strawberry, PAL was related to the anthocyanin accumulation, and anthocyanin is a kind of flavonoid which has lots of health care functions as well as an effect in modification of color [24, 50]. As shown in Fig. 2, the color of *E. ferox* seed gradually deepened during the growth and development. This phenotypic change may be regulated by the associated secondary metabolite. Whereas *CcPAL1*, *CcPAL2* and *CcPAL3* were verified with the highest expression in the early stage of bean and pericarp in *Coffea canephora* [51]. The most likely reason was that the synthetic metabolites were different in the growth and development of plants. In fact, the types of metabolite were very rich due to the complexity of the metabolic pathway. Two *PALs* in *Populus tremuloides*, *PtPAL1* was associated with condensed tannin metabolism and *PtPAL2* was involved in monolignol biosynthesis, of which, tannin has remarkable biological and pharmacological activities [52, 53]. In coffee bean, *CcPAL1* and *CcPAL3* were linked with the accumulation of cholorogenic acids (CGA), whereas *CcPAL2* may contribute more importantly to flavonoid accumulation [51]. As a traditional herb, *E. ferox* seeds are available for human health, so the accumulation of secondary metabolites is closely related to the growth and development of *E. ferox* seeds. Lignin, as another vital secondary metabolite, is quite crucial for structural integrity of the cell wall and strength and stiffness of the stem [54]. All of the *AtPAL1*, *AtPAL2* and *AtPAL4* were associated with lignin biosynthesis in *Arabidopsis thaliana* [18]. As shown in Fig. 6, there are multiple methods to synthesize lignin during the *E. ferox* seeds development. In addition to *PAL*, *CYP84A1* (F5H) involved in biosynthetic pathway of lignin. The Fig. 2 of four stages in *E. ferox* seeds shown that, the stiffness of seeds was enhanced. This phenotypic change may be closely related to the accumulation of lignin. During the seed development in wheat, silencing of *TaCYP78A3* led to the decrease in wheat seed size and cell proliferation in wheat seed coats, so *TaCYP78A3* gene was proved that it can influence the size of seed [26]. The fruit and seeds of *E. ferox* became obviously larger at the four development stages (Fig. 2). The example in wheat seed can help understand the cellular basis of genes influencing the *E. ferox* seed development.

For all of the DEGs in *E. ferox* seed development, the number of DEGs between any two adjacent developmental stages (T1 vs. T2, T2 vs. T3, and T3 vs. T4) was far less than the number found in the T1 vs. T3, T1 vs. T4, and T2 vs. T4 comparisons (Additional file 5: Table S3). This clearly shows constant development in *E. ferox* seeds. In addition, there were only 21 DEGs in the T3 vs. T4 comparison (Additional file 5: Table S3), which indicated that accumulation of the substances tended to saturate at the later stages of seed development. Above all, the pattern of DEGs was coincident with the stages of *E. ferox* seed growth and development.

## Conclusions

In summary, we generated 313,844,425 clean reads from the 12 *E. ferox* seed cDNA libraries using the Illumina Hiseq 2000 platform. 85,006 unigenes with an N50 length of 1399 bp were obtained; of these, 17,769 unigenes were longer than 1 kb. Phenylpropanoid biosynthesis (129 genes annotated with 16 DEGs) involved in the synthesis of important secondary metabolites including flavonoids, lignins and alkaloid. PAL and P450 encoded genes related to this pathway played a vital role in the morphological changes and the accumulation of medicinal components. In the mature stage of *E. ferox* seed, 21 DEGs indicated that accumulation of the substances tended to saturate. And this pattern is consistent with the growth and development of *E. ferox* seed.

## Additional files

Additional file 1: **Figure S1.** KEGG taxonomy of differentially expressed genes. All of the unigenes were assigned to 127 pathways which were divided into five groups: cellular processes, environmental information processing, genetic information processing, metabolism, and organismal systems. (JPG 653 kb)

Additional file 2: **Figure S2.** A phylogenetic tree depicting the relationships among the beta-glucosidase genes in angiosperm. Forty-nine beta-glucosidase genes from angiosperms with Pinus contorta as a outgroup were used in this study. (JPG 2894 kb)

Additional file 3: **Table S1.** The assembled sequences of *Euryale ferox* were annotated by BLASTX against *Arabidopsis thaliana* TAIR10. (XLS 490703 kb)

Additional file 4: **Table S2.** The assembled sequences of *Euryale ferox* were annotated by BLASTX against rice 7.0 proteomes. (XLS 587887 kb)

Additional file 5: **Tables S3.** Differentially expressed genes (DEGs) identified in pairwise comparisons of developmental stages in *E. ferox* seeds. (DOCX 15 kb)

Additional file 6: **Table S4.** The FDR of differentially expressed genes (DEGs) identified in pairwise comparisons of developmental stages in *E. ferox* seeds. (DOCX 15 kb)

## Abbreviations

CGA: Cholorogenic acids
COG: Clusters of Orthologous Groups
CYP: Cytochrome P450
DEGs: Differentially expressed genes
F5H: Ferulate-5-hydroxylase
FC: Fold Change
FDR: False discovery rate
GO: Gene Ontology
KEGG: Kyoto Encyclopedia of Genes and Genomes
NJ: Neighbor-joining
NR: NCBI non-redundant protein sequences
Ns: Unknown bases
PAL: Phenylalanine ammonia-lyase
