# Supplementary figures and images for: Transcriptome sequencing and analysis during seed growth and development in Euryale ferox Salisb
Source: BMC Genomics. 2018 May 9;19:343. doi: 10.1186/s12864-018-4707-9 (PMC5944168; doi:10.1186/s12864-018-4707-9)

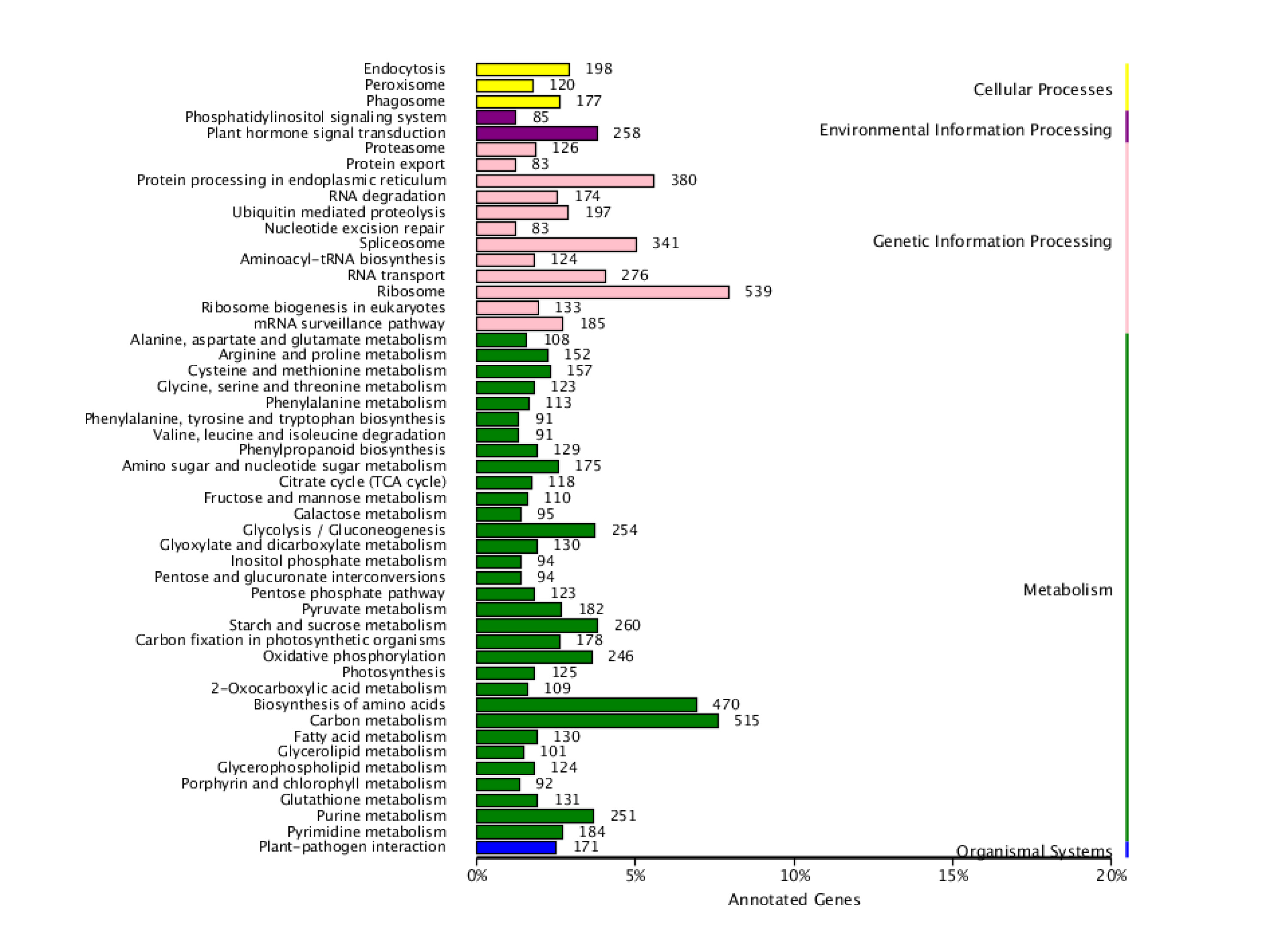

Supplement: Supplementary file 1 — Figure S1. KEGG taxonomy of differentially expressed genes. All of the unigenes were assigned to 127 pathways which were divided into five groups: cellular processes, environmental information processing, genetic information processing, metabolism, and organismal systems. (JPG 653 kb) [file 12864_2018_4707_MOESM1_ESM.jpg]

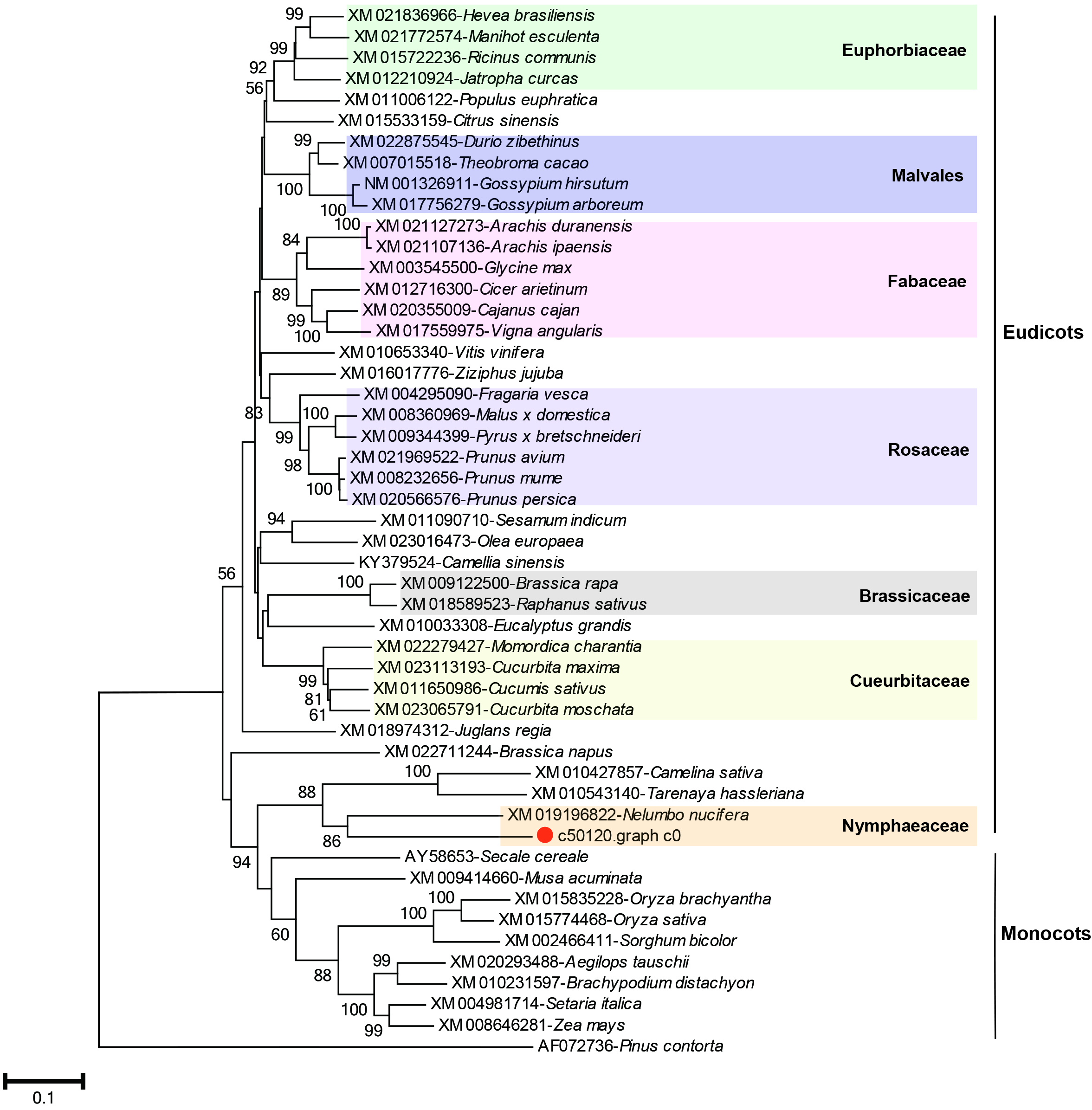

Supplement: Supplementary file 2 — Figure S2. A phylogenetic tree depicting the relationships among the beta-glucosidase genes in angiosperm. Forty-nine beta-glucosidase genes from angiosperms with Pinus contorta as a outgroup were used in this study. (JPG 2894 kb) [file 12864_2018_4707_MOESM2_ESM.jpg]
