# Supplementary material for: Transcriptome sequencing and analysis during seed growth and development in Euryale ferox Salisb
Source: BMC Genomics. 2018 May 9;19:343. doi: 10.1186/s12864-018-4707-9 (PMC5944168; doi:10.1186/s12864-018-4707-9)
Supplement: Supplementary file 5 — Tables S3. Differentially expressed genes (DEGs) identified in pairwise comparisons of developmental stages in E. ferox seeds. (DOCX 15 kb) [file 12864_2018_4707_MOESM5_ESM.docx]

**Table S3 | Differentially expressed genes (DEGs) identified in pairwise comparisons of developmental stages in *E. ferox* seeds.**

| Stage comparison | Total DEGs | Up-regulated | Down-regulated |
| --- | --- | --- | --- |
| T1 vs. T2 | 387 | 145 | 242 |
| T1 vs. T3 | 468 | 320 | 148 |
| T1 vs. T4 | 4509 | 2300 | 2209 |
| T2 vs. T3 | 47 | 47 | 0 |
| T2 vs. T4 | 1517 | 774 | 743 |
| T3 vs. T4 | 21 | 10 | 11 |
