# Supplementary material for: Transcriptome sequencing and analysis during seed growth and development in Euryale ferox Salisb
Source: BMC Genomics. 2018 May 9;19:343. doi: 10.1186/s12864-018-4707-9 (PMC5944168; doi:10.1186/s12864-018-4707-9)
Supplement: Supplementary file 6 — Table S4. The FDR of differentially expressed genes (DEGs) identified in pairwise comparisons of developmental stages in E. ferox seeds. (DOCX 15 kb) [file 12864_2018_4707_MOESM6_ESM.docx]

**Table S4 | The FDR of differentially expressed genes (DEGs) identified in pairwise comparisons of developmental stages in *E. ferox* seeds.**

| Gene identifier | FDR (T1 vs T2) | FDR (T1 vs T3) | FDR (T1 vs T4) | FDR (T2 vs T3) | FDR (T2 vs T4) | FDR (T3 vs T4) |
| --- | --- | --- | --- | --- | --- | --- |
| c44497.graph_c0 | - | - | 3.23E-20 | - | 2.00E-08 | 0.015174555 |
| c50120.graph_c0 | 0.000414818 | 0.272453083 | 0.09673629 | 0.003012097 | 0.53254095 | 0.403309519 |
| c17722.graph_c0 | - | 1.98E-07 | 1.53E-09 | 2.78E-06 | 3.54E-07 | 1 |
| c55946.graph_c2 | 0.52635143 | 5.78E-07 | 3.57E-05 | 0.000880056 | 0.009286417 | 1 |
| c41810.graph_c1 | 1.32E-07 | 6.91E-07 | 7.94E-34 | 0.369379614 | 0.048155434 | 1 |
| c53300.graph_c0 | - | 3.80E-05 | - | 0.001204541 | - | 0.691819713 |
